# Oxford Nanopore and Bionano Genomics technologies evaluation for plant structural variation detection

**DOI:** 10.1186/s12864-022-08499-4

**Published:** 2022-04-21

**Authors:** Aurélie Canaguier, Romane Guilbaud, Erwan Denis, Ghislaine Magdelenat, Caroline Belser, Benjamin Istace, Corinne Cruaud, Patrick Wincker, Marie-Christine Le Paslier, Patricia Faivre-Rampant, Valérie Barbe

**Affiliations:** 1 Université Paris-Saclay, INRAE, Etude du Polymorphisme des Génomes Végétaux EPGV, 91000 Evry-Courcouronnes, France; 2 Genoscope, Institut de biologie François-Jacob, Commissariat à l’Energie Atomique CEA, Université Paris-Saclay, Evry, France; 3 Génomique Métabolique, Genoscope, Institut François Jacob, CEA, CNRS, Univ Evry, Université Paris-Saclay, 91057 Evry, France

**Keywords:** Structural variations, Oxford Nanopore technologies, Bionano Genomics optical mapping, High molecular weight DNA, *Arabidopsis thaliana*

## Abstract

**Background:**

Structural Variations (SVs) are genomic rearrangements derived from duplication, deletion, insertion, inversion, and translocation events. In the past, SVs detection was limited to cytological approaches, then to Next-Generation Sequencing (NGS) short reads and partitioned assemblies. Nowadays, technologies such as DNA long read sequencing and optical mapping have revolutionized the understanding of SVs in genomes, due to the enhancement of the power of SVs detection.

This study aims to investigate performance of two techniques, 1) long-read sequencing obtained with the MinION device (Oxford Nanopore Technologies) and 2) optical mapping obtained with Saphyr device (Bionano Genomics) to detect and characterize SVs in the genomes of the two ecotypes of *Arabidopsis thaliana,* Columbia-0 (Col-0) and Landsberg *erecta* 1 (L*er*-1).

**Results:**

We described the SVs detected from the alignment of the best ONT assembly and DLE-1 optical maps of *A. thaliana* L*er*-1 against the public reference genome Col-0 TAIR10.1. After filtering (SV > 1 kb), 1184 and 591 L*er*-1 SVs were retained from ONT and Bionano technologies respectively. A total of 948 L*er*-1 ONT SVs (80.1%) corresponded to 563 Bionano SVs (95.3%) leading to 563 common locations. The specific locations were scrutinized to assess improvement in SV detection by either technology. The ONT SVs were mostly detected near TE and gene features, and resistance genes seemed particularly impacted.

**Conclusions:**

Structural variations linked to ONT sequencing error were removed and false positives limited, with high quality Bionano SVs being conserved. When compared with the Col-0 TAIR10.1 reference genome, most of the detected SVs discovered by both technologies were found in the same locations. ONT assembly sequence leads to more specific SVs than Bionano one, the latter being more efficient to characterize large SVs. Even if both technologies are complementary approaches, ONT data appears to be more adapted to large scale populations studies, while Bionano performs better in improving assembly and describing specificity of a genome compared to a reference.

**Supplementary Information:**

The online version contains supplementary material available at 10.1186/s12864-022-08499-4.

## Background

Structural variations (SV) are genomic variations involving segments of DNA from 50 bases to several megabases. SVs consist of unbalanced rearrangements such as copy number variations (CNV) including insertions/deletions (Indels) and presence/absence variations (PAV), and balanced events like inversions and translocations [1–4]. Several mechanisms explain the SVs formation, such as recombination errors generated by non-homologous end- joining and non-allelic homologous recombination, genome duplication and transposition [1, 2]. The structural variations in humans were largely studied and Ho et al. reviewed the impact of the SVs in human diseases [4]. In plants, it has been shown that the SVs play a key role in the evolution of genomes and are responsible for phenotypic variations by affecting Transposable Elements (TEs) and genes [3, 5–8]. In particular, SVs were found in stress related and resistance genes [9–13], leading to local adaptation [14, 15], or linked to other traits of agronomical interest such as tomato fruit flavor, rice grain size or poplar wood formation [16–18].

Nowadays, the identification of SVs contributes to the construction of the pangenome reference sequence or super pangenome [19, 20]. This new approach to build a reference will better reflect the genetic diversity of the species, and at the same time expand the understanding of genome evolution, as well as enhance the knowledge on adaptive traits [21–25].

The development of new sequencing technologies has boosted studies of SVs present in a genome, which were detected until recently only by Comparative Genomic Hybridation (CGH) arrays or single nucleotide polymorphism (SNP) [26–29]. The 3rd generation sequencing offers new opportunities to identify SVs at a larger scale with two approaches. One approach is based on linked short reads, as in 10x Genomics and Hi-C approaches [30], and the second by generating long reads, as proposed by Pacific Biosciences [31] and Oxford Nanopore Technologies (ONT) [32, 33]. These approaches provide access to complex regions, increasing their uses to improve genome assemblies and to detect structural variations in human [4, 34–37], in *Arabidopsis thaliana* ecotypes [24, 38, 39] and T-DNA insertion lines [40, 41] and in other plants [42–44]. In parallel, a technology based on physical map and developed by Bionano Genomics [45], generates information on very large DNA molecules. These maps, named optical maps, are frequently generated to improve and validate sequencing assembly, to detect SVs in animals genomes [36, 46–49] and more recently in plants [7, 42, 43, 50]. These 3rd generation technologies with combination possibilities made possible the identification of genetic rearrangements between individuals at intra specific levels [50, 51].

Comparisons between sequencing technologies or SV detection software are no longer uncharted territory [24, 36, 38, 52]. However, the comparison of ONT and Bionano was only performed in animals (Chimpanzee [49] and Drosophila [53]), but not yet in plants. Here, we investigated the genomes of two most studies ecotypes of *A. thaliana* (Col-0 and L*er*-1) obtained by both ONT and Bionano optical maps to compare the advantages of these two fundamentally different technologies, sequencing-based and physical map, to provide information on detection and characterization of SVs in plants.

## Results

### ONT sequencing and genome assembly

The ONT sequences of *Arabidopsis thaliana* ecotypes, Columbia (Col-0), here named as Evry.Col-0 and Landsberg *erecta* 1 (L*er*-1), here named as Evry.L*er*-1, were cleaned using the correction and trimming steps of Canu assembler [54]. A total of 9.8 Gb (N50 = 12.7 kb, 75X coverage) and 6.1 Gb (N50 = 16.5 kb, 47X coverage) were obtained for Evry.Col-0 and Evry.L*er*-1, respectively (Additional file 1: Tables S1 and S2).

**Fig. 1:**
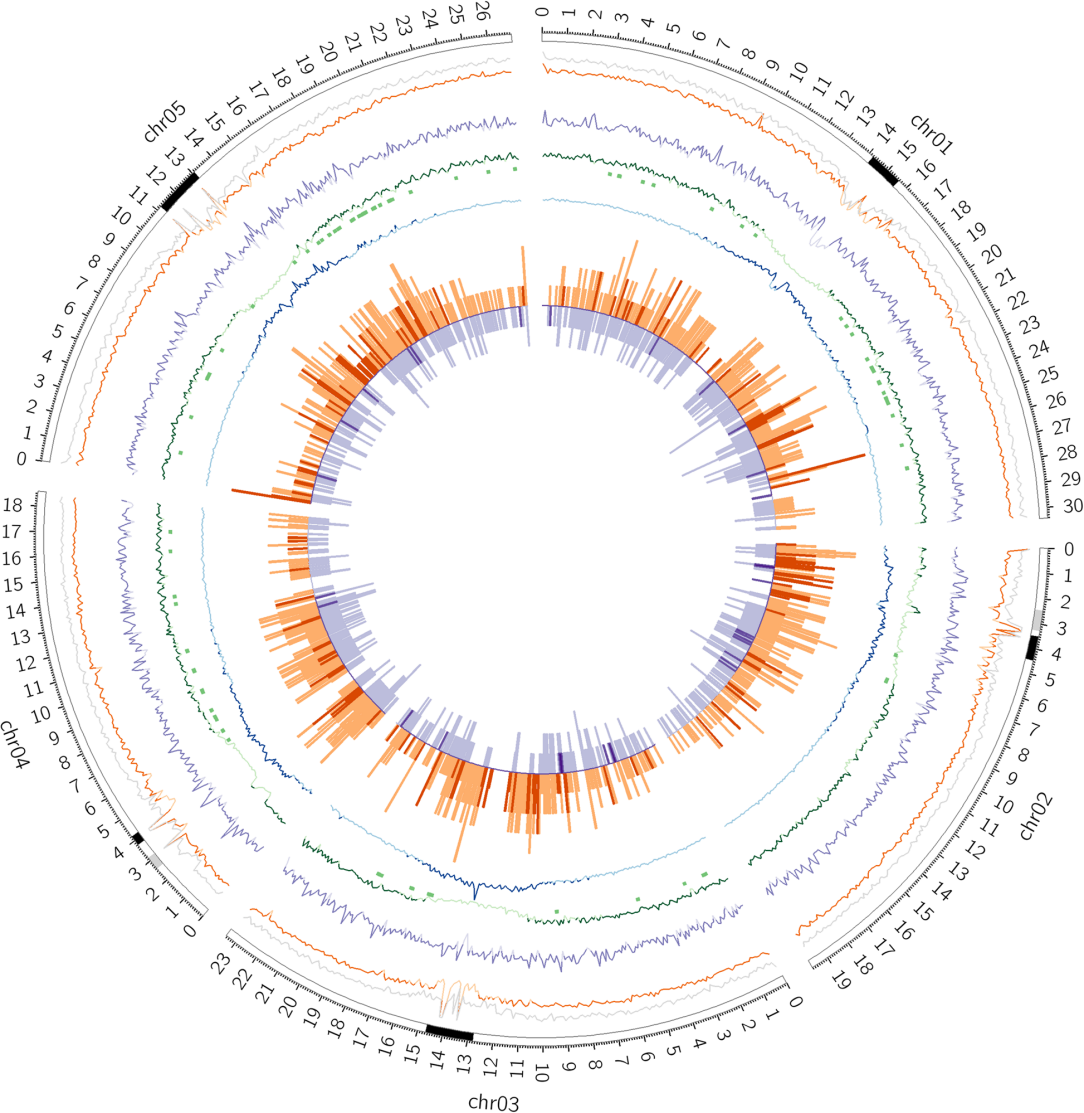
Circos visualization of Evry.L*er*-1 SVs landscape. All comparisons were performed against the Col-0 TAIR10.1 reference genome per 100kb bins. From external to internal layer (Circle1 to Circle7): Circle1: Col-0 TAIR10.1 chromosomes (ticks every 100 kb): black and light grey rectangles represent centromeric and NOR regions respectively; Circle2: Average mapping coverage for Evry.Col-0 ONT reads (grey line) and Evry.L*er*-1 ONT reads (orange line) with dark orange if coverage > 46X; Circle3: DLE-1 label density as purple line (dark purple if density > 18 label per 100 kb); Circle4: Genes density as green line (dark green if density > 23), NLR Genes [58] indicated as green rectangles; Circle5: TEs density as blue line (dark blue if density > 58); Circle6: ONT SVs occurrences as orange outward bars (dark orange bars represent ONT- specific SVs); Circle7: Bionano SVs occurrences as purple inward bars (dark purple bars represent Bionano-specific SVs)

Cleaned Evry.L*er*-1 ONT reads were aligned against the L*er* reference genome with Minimap2 to estimate ONT data completeness [38, 55]. In total, 98.9% of the L*er* reference genome was covered by the ONT Evry.L*er*-1 reads. The cleaned Evry.L*er*-1 reads were also mapped against the Col-0 TAIR10.1 reference genome achieving 95.2% of total genome coverage (Additional file 1: Table S3) [56]. Samtools *depth* tool [57] was then used on the Evry.L*er*-1 ONT reads mapping against the Col-0 TAIR10.1 reference genome to estimate the coverage at each position. The average coverage of 100 kb windows was 46.9X, with depth fluctuations in centromeric regions (Fig. 1).

To select the assembler that could produce a better output for our data, de novo assemblies for Evry.Col-0 and Evry.L*er*-1 were performed with Canu [54], RA [59] and SMARTdenovo (SDN, [60]). Based on general statistics (assembly size, contig number, N50 size), SMARTdenovo software generated better assemblies for both ecotypes compared to Canu or RA. (Additional file 1: Tables S4 and S5). Indeed, the SDN assemblies resulted in 79 contigs for Evry.Col-0 (cumulative size =117 Mb, N50 = 12.5 Mb with L50 = 5 contigs) and 101 contigs for Evry.L*er*-1 (cumulative sizes = 117 Mb, N50 = 10.7 Mb with L50 = 5 contigs). Assemblies using RA were more fragmented and chimeric contigs were identified with Canu assembler after MUMmer alignments on the reference chromosomes (Additional file 2: Figs. S1A-C and S2A-C). For all assemblers tested, centromeric regions were covered by many small contigs. These results were also supported by the alignments of the Evry.Col-0 and Evry.L*er*-1 assemblies on the respective reference chromosomes Col-0 TAIR10.1 and L*er*. The SDN were used to perform the subsequent SV analyses.

### Optical maps generation

Genomic DNA was labeled using staining protocol with DLE-1 enzyme according to the manufacturer’s protocol. One run per ecotype on the Saphyr device was performed resulting to 577.5 Gb and 610.9 Gb of molecules for Evry.Col-0 and Evry.L*er*-1 respectively. Molecules larger than 150 kb were selected leading to about 600-fold final coverage based on the theoretical 130 Mb *Arabidopsis* genome size (Additional file 1: Tables S6 and S7). A total of 17 and 14 optical maps with N50 of 14.6 Mb and 14.7 Mb were generated for Evry.Col-0 and Evry.L*er*-1 respectively, leading to a genome size of 125 Mb for both ecotypes (Additional file 1: Tables S8 and S9).

The average label density of the Evry.L*er*-1 optical maps was estimated at 18.47 per 100 kb (Additional file 1: Table S7). However, the DLE-1 density decreases in the centromeric regions due to molecule depth diminution and optical map breaks (Fig. 1, Additional file 2: Fig. S3A-E).

### Structural variations detection

Structural variations detections were performed independently using the ONT and Bionano technologies data and were carried out in two ways: 1) Evry.L*er*-1 versus Col-0 TAIR10.1 reference genome and 2) Evry.Col-0 versus L*er* reference genome. The different types of structural variations detected in our study are described in Additional file 2: Fig. S4. We observed that general SVs characteristics (number, types and location) are similar in both ways, then only SV detection results from the Evry.L*er*-1 assembly and optical maps against the Col-0 TAIR10.1 reference genome will be presented in detail. Description of SVs detected by comparing the SDN assembly and optical maps Evry.Col-0 with L*er* reference genome are provided in Additional file 1: Tables S10-S14 and Additional file 2: Fig. S5A-E.

The sequence comparison of Evry.L*er*-1 assembly to Col-0 TAIR10.1 reference genome using MUMmer *show-diff* utility [61] revealed 2186 potential SVs. A total of 119 SVs, called reference sequence junction (SEQ), break (BRK) and jump (JMP), found in centromeric, telomeric and nearby rDNA clusters, were considered to correspond to unresolved assembly regions into Evry.L*er*-1 assembly compared to Col-0 TAIR10.1 reference genome and were filtered out (Additional file 1: Table S15).

**Fig. 2:**
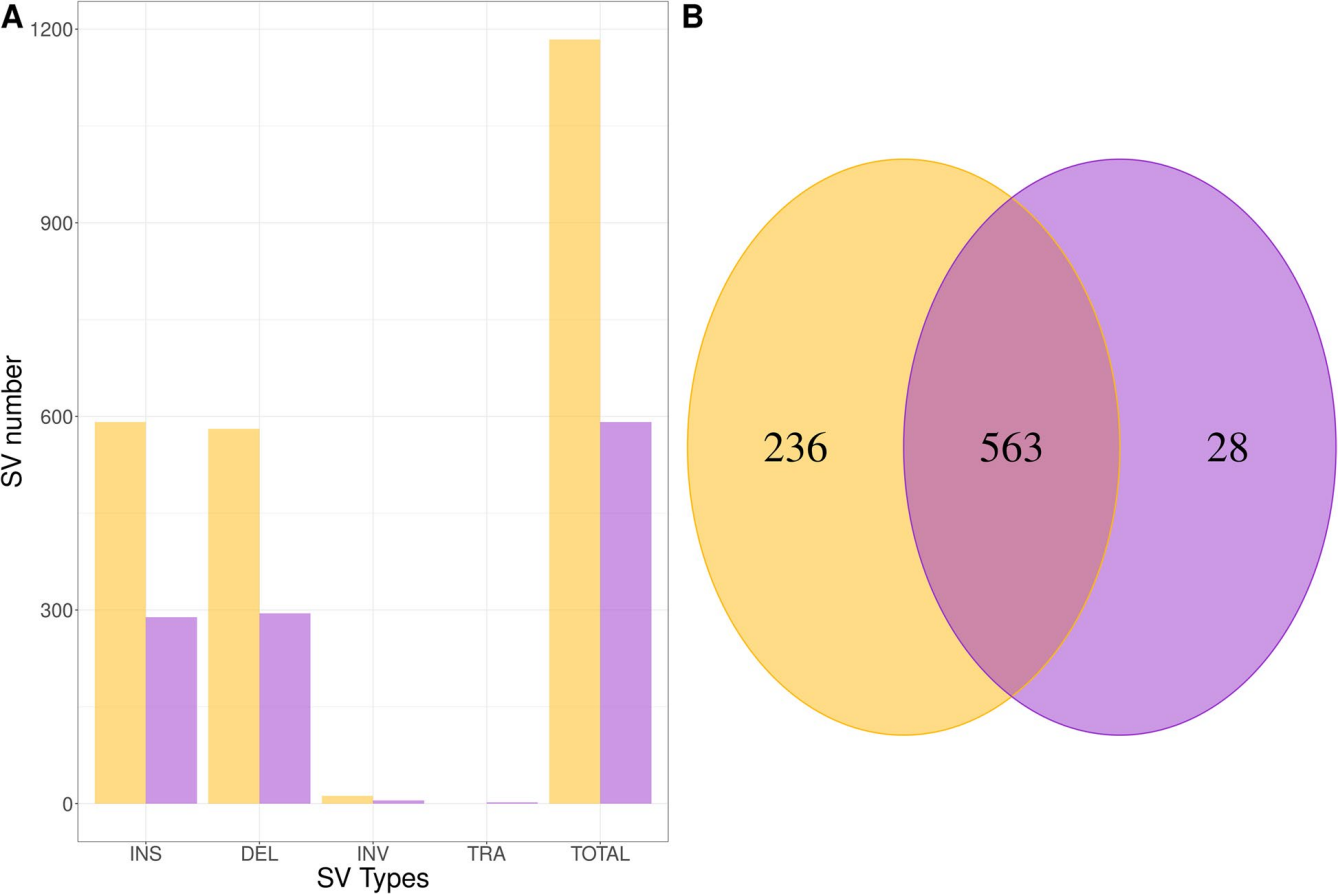
Number of Evry.L*er*-1 structural variations detected by ONT and Bionano against the Col-0 TAIR10.1 reference genome and overlaps in locations between the two technologies. The bars and circles colored in orange and purple correspond respectively to the ONT and Bionano technologies. **A** Barplot of SV number for insertions (INS), deletions (DEL), inversions (INV), translocations (TRA) and all SVs (TOTAL) obtained using ONT and Bionano technologies. **B** Venn diagramm of common and specific locations detected by ONT and Bionano technologies

The estimation of the ONT error sequencing rate was 4.0 and 4.9% for the Evry.Col-0 and Evry.L*er*-1 of the trimmed corrected sequences respectively. Even if these error sequencing rates are inferior than previously described [62], to avoid false positive SV detection and to be comparable to Bionano technology, a filter on query ONT structural variations size (> 1 kb, SV detection size limit for high quality Bionano technology) was applied. On the 1184 SVs > 1 kb (54.2%), 591 insertions (INS), 581 deletions (DEL), 12 inversions (INV) were detected but no duplication (Table 1 and Fig. 2A).

**Table 1:** Characteristics of Evry.L*er*-1 ONT and Bionano SVs, obtained after alignment against Col-0 TAIR10.1 reference genome

Technology	ONT				Bionano				
SV type	INS	DEL	INV	TOTAL	INS	DEL	INV	TRA	TOTAL
SV > 1 kb (%)	591 (49.9)	581 (49.1)	12 (1.0)	1184	289 (48.9)	295 (49.9)	5 (0.8)	2 (0.4)	591
Cumulated Size	3.4	4.0	0.3	7.7	2.9	2.3	1.6	0.4	7.2
Median Size	3358	3446	17,012	3455	4383	4296	166,007	189,454	4383
Average Size	5724	6801	26,865	6467	10,021	7885	310,470	189,454	12,104

Cumulated sizes are in Mb, Median and Average sizes in bp

A 5 Mb insertion in the Evry.L*er*-1 assembly was detected on Chr3 Col-0 TAIR10.1 reference genome (14,272,986..14284724) due to a detection error of MUMmer in a complex region associated with a rDNA cluster. Thereby, this insertion was removed from the final data and not considered in the result. The Evry.L*er*-1 ONT median size of the structural variations was 3455 bp and the cumulated size of 7.7 Mb. The SVs were equally distributed in size and number between INS and DEL. The INV categories had higher median and average sizes than INS and DEL. With a cumulated size of 0.3 Mb, INV represented 3.9% of the ONT variation size (Table 1). Structural variations were detected on all chromosomes, with a preferential location on chromosome arms and with no confident SV on the Chr1, 3 and 4 centromeres (Fig. 1).

**Fig. 3:**
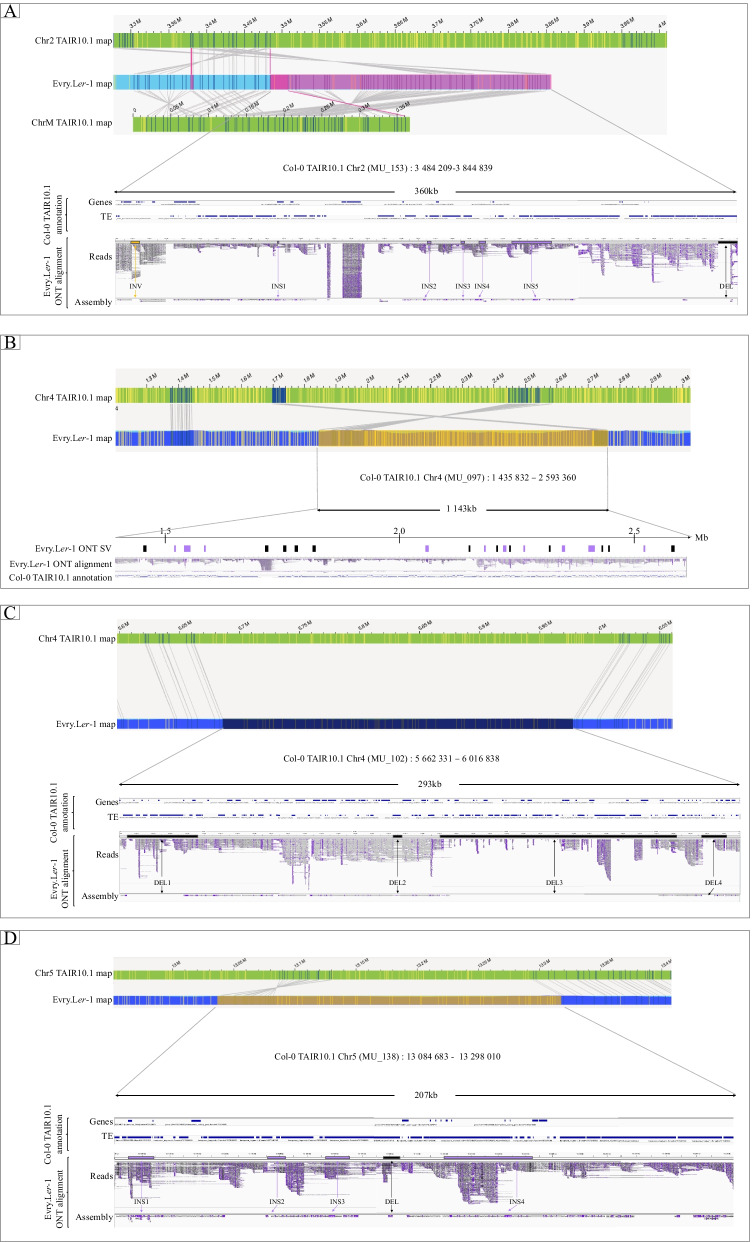
Focus of large structural variations (MU) located in complex locations. For each location, optical maps are colored in green for the Col-0 TAIR10.1 reference maps (ChrM for mitochondrial chromosome map) and light blue for Evry.L*er*-1 maps. Consistent DLE-1 enzyme label between reference and Evry.L*er*-1 maps are represented by dark blue bars with grey links between the genome maps. Inconsistent DLE-1 enzyme label are shown by yellow bars on the two genome maps. The purple bars represent the insertion events on the Evry.L*er*-1 maps / read / assembly, and the black, yellow and pink bars correspond to deletions, inversions and translocations respectively. Araport11 annotation of the Col-0 TAIR10.1 reference (Genes and TE) and IGV view of the Evry.L*er*-1 trimmed ONT reads and SMARTdenovo contigs minimap alignments are also presented. **A** Bionano Chr2 Evry.L*er*-1 translocations against Col-0 TAIR10.1 reference genome (MU_153). **B** Bionano Chr4 Evry.L*er*-1 extra-range size inversion against Col-0 TAIR10.1 reference genome (MU_097). **C** Bionano Chr4 Evry.L*er*-1 large deletion against Col-0 TAIR10.1 reference genome (MU_102). **D** Bionano Chr5 Evry.L*er*-1 inversion against Col-0 TAIR10.1 reference genome (MU_138)

Optical maps construction and SVs detection based on physical maps comparison was carried out on the Bionano Solve™ interface (Bionano Genomics, version 3.3). A total of 797 SVs were highlighted by comparing Evry.L*er*-1 optical maps to in silico Col-0 TAIR10.1 reference genome labeling with DLE-1(Additional file 1: Table S15). When Bionano Solve tools detected one SV embedded in a second one, the largest SV was kept. This case was found on two Chr1 independent locations (INS:19432310..19468513 and DEL:24688666..24736849). A 1 kb size filter was applied on the Bionano SVs, which was equivalent to remove deletions and insertions with a Bionano quality score < 10 (defined as poor quality by the manufacturer) (Additional file 1: Table S16). Additionally, on Chr2, the INV SV (3,433,371..3490731) with no quality score was discarded. Thereby, 591 SVs representing 74.2% of total optical map Evry.L*er*-1 SVs were further considered in this analysis. INS and DEL types constituted the main part of the optical map Evry.L*er*-1 SVs (48.9 and 49.9% of the SVs respectively), the remaining 1.2% corresponding to translocations (TRA) and INV (Table 1 and Fig. 2A). Median SVs size was 4383 bp and SVs cumulated sizes represented 7.2 Mb of the genome. The TRA and INV types corresponded to nearly one third (2.0 Mb) of the structural variations cumulated size. In our study, the translocation type was only detected using the Bionano assembly (Table 1 and Fig. 2A). The two Evry.L*er*-1 TRA were located on Chr2 (3,378,844..3397121; 3,484,209..3844839) (Fig. 3A and Additional file 2: Fig. S3B). The largest SV identified was a 1.1 Mb Evry.L*er*-1 INV located on Col-0 TAIR10.1 reference genome Chr4 (1,435,832..2593360) (Fig. 3B and Additional file 2: Fig. S3D). SVs were distributed preferentially along the chromosome arms and their detection was limited in centromeric regions due to decrease in labeling in these regions (Fig. 1).

### SVs comparison

SVs comparison was based on their absolute start- and end-positions on the Col-0 TAIR10.1 reference genome. We considered that structural variations locations were comparable in both technologies when their locations on Col-0 TAIR10.1 reference genome overlapped by at least 1 bp.

SVs comparison metrics are presented in Table 2 and the numbers of overlapping locations in Fig. 2B. A total of 563 common locations were identified representing 948 (80.1%) of Evry.L*er*-1 ONT SVs and 563 (95.3%) of optical map Evry.L*er*-1 SVs. The cumulated sizes of these common SVs were respectively 5.9 Mb and 6.9 Mb for ONT and Bionano detection representing 5.3% of the size of the Col-0 TAIR10.1 reference genome (based on 130 Mb) for ONT and 4.5% for Bionano. ONT SVs tended to be smaller than Bionano SVs (Table 2, Additional file 1 Tables S17 and S18).

**Table 2:** Characteristics of Evry.L*er*-1 ONT and Evry.L*er*-1 optical map SVs identified in common and specific Col-0 TAIR10.1 reference locations

Technology	ONT		Bionano	
	Common	Specific	Common	Specific
Locations	563	236	563	28
SVs > 1 kb (%)	948 (80.1)	236 (19.9)	563 (95.3)	28 (4.7)
Min Size	1003	1003	1034	1017
Max Size	87,533	347,239	1,143,224	166,007
Cumulated Size	5.9	1.8	6.9	0.3
Median Size	3759	2656	4456	1374
Average Size	6221	7453	12,171	11,104

Cumulated sizes are in Mb, and all other sizes in bp

**Fig. 4:**
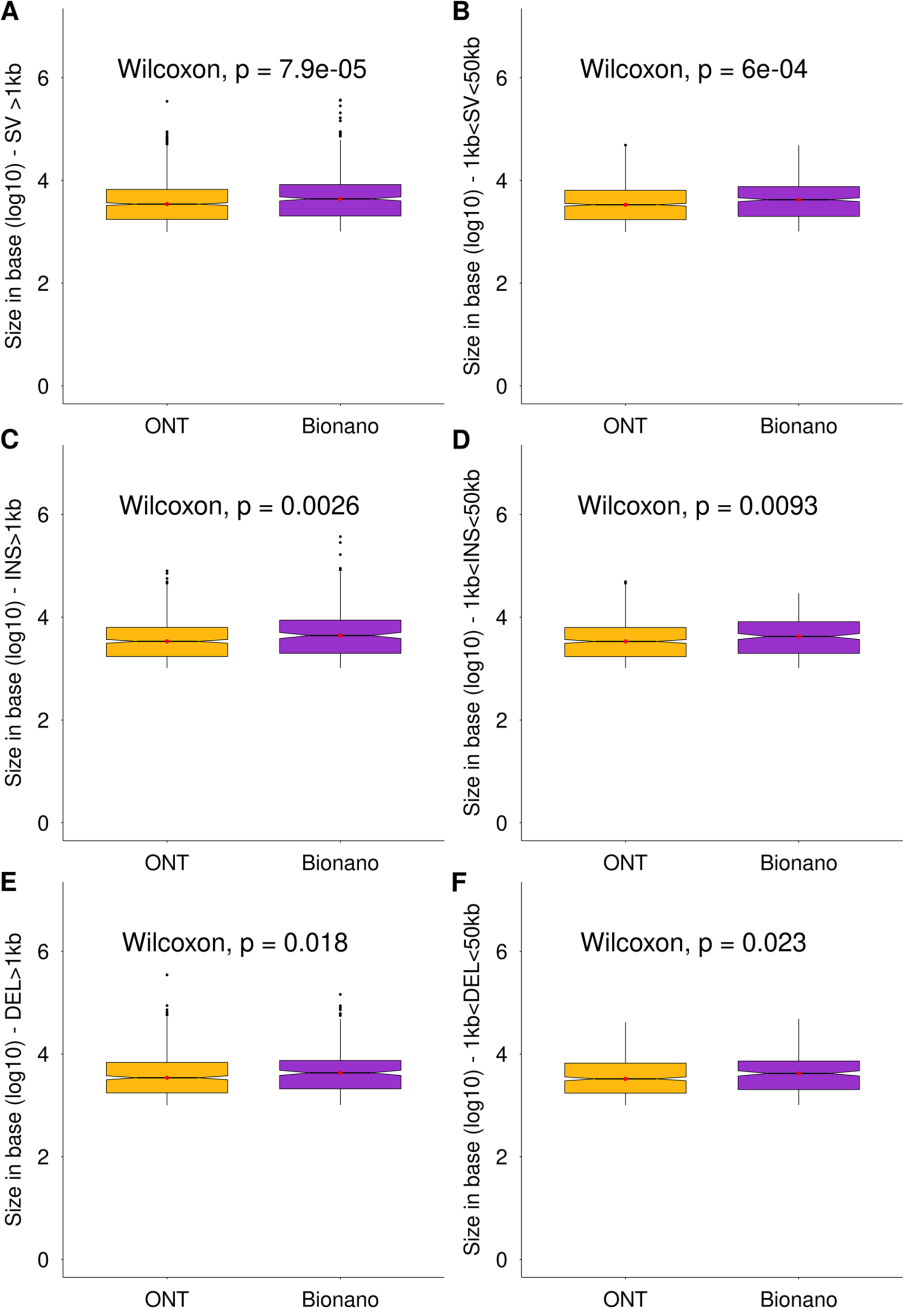
Size distribution and median comparison of ONT and Bionano SV. All p-values were obtained with a two-sided oriented Wilcoxon rank-sum test. Hypothesis H0 was “median of ONT SV size is less than Bionano one”. ONT and Bionano boxplots are colored in orange and purple respectively. Medians are represented by red dots. **A** Boxplot of ONT (*n*=1184) and Bionano (*n*=591) SV>1kb. **B** Boxplot of ONT (*n*=1169) and Bionano (*n*=573) 1kb<SV<50kb. **C** Boxplot of ONT (*n*=591) and Bionano (*n*=289) INS>1kb. **D** Boxplot of ONT (*n*=588) and Bionano (*n*=282) 1kb<INS<50kb. **E** Boxplot of ONT (*n*=581) and Bionano (*n*=295) DEL>1kb. **F** Boxplot of ONT (*n*=571) and Bionano (*n*=288) 1kb<DEL<50kb

To compare the median sizes of the ONT and Bionano variations (> 1 kb), we made notched boxplots including or not the large events (> 50 kb) (Fig. 4). Using the oriented Wilcoxon rank-sum test as it was performed by Dixon at *al.* (2018), *p*-values of the tests are all less than the significance level alpha = 0.05 therefore the median sizes of SV ONT are significantly smaller than the median sizes of SV Bionano. In addition, the sizes of the medians of all insertions and those of deletions detected using the Bionano technology were respectively 30.5 and 24.6% larger than with ONT. This last point is related to the fact that we applied a filter for ONT SVs (> 1 kb), thus increasing the median sizes for all categories.

To go further, SVs identified by ONT and Bionano technologies were assigned to a two letters svID code. The first letter used for ONT SVs and the second for Bionano SVs, leading to common (svID UU and MU) and specific (svID UN and NU) locations (with “U” for “Unique location”, “M” for “Multiple locations” and “N” for “No location”, Additional file 1: Tables S17 and S18).

Among the 563 common regions, 410 (72.8%) coincided with svID UU, i.e. one ONT structural variation corresponding to one SV Bionano. For 364 (88.8%) of the sv ID UUs the overlap of these locations was 100% and for 30 (7.3%) greater than 50%. Only 16 (3.9%) svID UUs had less than 50% overlap (Additional file 1: Table S17). Moreover, 405 (98.8%) of the svID UU SVs have “conforming” type (i.e. have the same type) (Additional file 1: Table S17) and five svID UU (1.2%) were identified as deletions by ONT and insertions by Bionano technologies (svID UU_035, UU_038, UU_057, UU_073, UU_358; Additional file 1: Tables S17 and S18).

The remaining 153 (27.2%) common locations corresponded to 538 Evry.L*er*-1 ONT SVs (56.8% of commons ONT SVs) related to 153 Evry.L*er*-1 optical map SVs (27.2% of commons Bionano SVs) (Table 3).

**Table 3:** Characteristics of the svID MU identified in ONT and Bionano SVs

Technology	ONT	Bionano
Location	153	153
Number	538	153
Min Size	1010	1253
Max Size	87,533	1,143,224
Cumulated Size	3.9	4.4
Median Size	4236	9523
Average Size	7406	28,734

svID MU corresponds to locations where Multiple ONT SVs overlap a Unique Bionano SV location. Cumulated sizes are in Mb, and all other sizes in bp

These structural variations had a svID MU ranging to 2 (representing 59.5%) to 22 ONT SVs for one Bionano SV. The cumulative size of this SVs category was approximately 4 Mb for both technologies although the number of ONT variants is 3.5 times higher than in Bionano (538 vs 153). The size distribution of these SVs started from 1 kb (due to the filter applied) to 87 kb and 1.1 Mb respectively for ONT and Bionano. Furthermore, Bionano median and average sizes were 2 and 4 fold larger respectively. Unlike the svID UU, the type of the svID MU was “conforming” for only 68 (44.5%) locations of which 58 (85.3%) corresponded to 2 ONT SVs for 1 Bionano SV. The remaining 10 (14.7%) locations comprised 3 or 4 ONT SVs for one Bionano SV.

The largest ONT SV was included in a complex SV (svID MU_102) consisting of four contiguous deletions located on Chr4. These four deletions coincided with one Evry.L*er*1 optical map deletion (Fig. 3C, Additional file 1: Tables S17 and S18). The largest Evry.L*er*1 optical map SV (svID MU_097) was an inversion on Chr4 of 1,143,224 Mb overlapping 22 Evry.L*er*-1 ONT SVs (corresponding to INS and DEL) (Fig. 3B, Additional file 1: Tables S17 and S18). To enrich the list of the large (> 50 kb) Bionano SV, the svID MU_138, an INV of 207 kb was located on the Chr5 and covered 5 ONT structural variations (4 INS and 1 DEL) (Fig. 3D, Additional file 1: Tables S17 and S18).

Specific locations were more abundant with the ONT technology (236 SVs - svID UN, SV detected with ONT only - 19.9%) than with Bionano (28 SVs - svID NU, SV detected with Bionano only - 4.7%) leading to a cumulated size of 1.8 Mb and 0.3 Mb respectively, and with a median size twice larger (2656 bp for Evry.L*er*-1 ONT SVs vs 1374 bp for Bionano Evry.L*er*-1 optical map SVs). The distribution of the specific Evry.L*er*-1 ONT SVs onto the Col-0 TAIR10.1 reference chromosomes led to a clear trend to locate on NOR and centromeres (Fig. 1). The largest specific ONT variant was located on Chr3 and corresponded to a DEL (svID UN_124, Additional file 1: Table S17). The largest specific Bionano SV was spotted on the Chr3 and corresponded to an INV type (svID NU_017, Additional file 1: Table S18, Additional file 2: Fig. S3C). A focus on the TRA located on Chr2 revealed 18.2 kb specific Evry.L*er*-1 optical map SV (svID NU_007), close to the second TRA of 360 kb (MU_153). This last TRA (MU_153) coincided with seven SV events (1 INV, 5 INS and 1 DEL) in the Evry.L*er*-1 assembly (Fig. 3A, Additional file 1: Tables S17 and S18).

Using Araport11 annotation of the Col-0 TAIR10.1 reference genome (The Arabidopsis Information Resource – TAIR), a comparison using only Evry.L*er*1 ONT SVs is shown in Table 4. Since the Evry.L*er*-1 optical map events represented a large-scale observation, they were not taken into account in this analysis. A total of 893 (75.4%) out of 1184 Evry.L*er*1 ONT SVs overlapped TE features, of which 579 also overlapped genes. Only 291 (24.6%) Evry.L*er*-1 ONT SVs were located outside a TE feature, overlapping genes [125 (10.6%)] or not [166 (14.0%)] (Table 4). Focusing on Evry.L*er*-1 ONT specific SVs, their overlap with the Col-0 TAIR10.1 reference annotation showed a similar percentage compared to the common SVs.

**Table 4:** Evry.L*er*-1 ONT SVs (> 1 kb) overlapping Col-0 TAIR10.1 reference genes and TEs annotation features

Features		noTE		TE		TOTAL (%)
		noGENE	GENE	noGENE	GENE	
Common SV	UU	79	59	93	179	410 (34.6)
	MU	59	38	150	291	538 (45.5)
Specific SV	UN	28	28	71	109	236 (19.9)
SV number (%)		166 (14.0)	125 (10.6)	314 (26.5)	579 (48.9)	1184
TOTAL (%)		291 (24.6)		893 (75.4)		1184

To better characterize the genes affected by ONT SVs in common locations, a GO-terms overrepresentation test was performed with the PANTHER’s tool [63] available on TAIR website (https://www.arabidopsis.org/tools/go_term_enrichment.jsp). Among the 1764 genes identified in common locations, 47.2% (832) genes were uniquely assigned to a GO term and used in PANTHER (Additional file 1: Tables S19 and S20). Overrepresentations in defense response and ADP-binding terms were detected (Additional file 1: Table S21), but no enrichment for GO-terms in genes in specific ONT locations was highlighted (Additional file 1: Tables S22-S24).

A summary of the main comparison criteria between the two technologies is presented in Table 5. It appears that the ONT and Bionano technologies (with DLS labeling) were equally effective in detecting SVs of less than 50 kb and those in gene regions. In our study, Bionano was more efficient on large events while additional analyzes for the detection of these variations with ONT are necessary.

**Table 5:** Summary of comparisons of ONT assembly and Bionano Optical mapping (DLS labeling) for SV detection

Technology	ONT	Bionano
**Assembly/Optical map obtention**	√√	√√
**1 kb < SV < 50 kb**		
Deletion	√√	√
Insertion	√√	√
Inversion	√	√√
Translocation	NA	√
**SV > 50 kb**		
Deletion	√	√√
Insertion	√	√√
Inversion	–	√√
Translocation	NA	√√
**SV location**		
Genic	√√	√√
Complex	√	√√
**Acces to the SV sequence**	√√	NA
**Bioinformatic skills**	√	√√
**SV visualization**	√	√√

√√ Robust detection or user friendly. √ Detection depending on variables such as coverage, contig length and label density or need of bioinformatic skills. NA: Not available; − Undetectable

## Discussion

Herein, we compare the performance of Oxford Nanopore and Bionano Genomics technologies for structural variation detection. For this, we performed long read sequencing and optical mapping of two *A. thaliana* ecotypes, namely Columbia-0 (Col-0) and Landsberg *erecta* 1 (L*er*-1). Long read de novo assemblies were constructed using three different assemblers and optical maps were assembled with Bionano Solve tools. Structural variations detected using the Col-0 TAIR10.1 [56] and L*er* [38] genomic sequences as references, were described and compared to each other, to reveal the relative strengths of the two technologies in detecting SVs.

### Assemblies based on ONT and Bionano data for SV analyses

To obtain the best assembly based on only long reads data we used three different assemblers. After comparison of assembly metrics, calculation time and collinearity against reference genomes, SDN provided the best assembly even if some collinearity breaks were observed, especially in centromeric regions. The metrics of Evry.Col-0 and Evry.L*er*-1 SDN assemblies were comparable to such assemblies in previous studies [24, 38, 39, 64] but remained underestimated.

Continuous improvement in protocols and new developments in genome assembly strategies and algorithms resulted in a higher quality of genomic sequences used in subsequent analyses. Previously published Bionano *A. thaliana* optical map (KBS-Mac-74 genome [39]) used a BspQI staining protocol for labeling, generating about 10 times more maps to cover the entire genome of the KBS-Mac-74 ecotype than in our study (DLE-1 Bionano staining protocol), highlighting enhancement in Bionano’s protocol. In addition, no optical map was previously available for the Columbia (Col-0) and Landsberg *erecta* 1 (L*er*-1), making our map assemblies especially valuable for further studies.

Our high quality optical map allowed us to define centromeric and nucleolar organizer regions (NOR), despite lower molecules density and even if label concordance loss were observed between Evry.L*er*-1 maps compared to the Col-0 TAIR10.1 in silico reference maps. Moreover, fluctuations in ONT coverage density and accumulation of repetitive alignments in the same regions are reinforcing evidence of the approximate locations of the centromeres and NOR. However, we identified several misassemblies in the course of our SVs analyses between the Evry.L*er*-1 SDN assembly and Col-0 TAIR10.1 reference genome, highlighting how difficult it can be to get a reliable assembly, and thus detecting SVs, in these complex regions.

### SV detection and comparison between the two technologies

We compared structural variations in Evry.L*er*-1 and the reference genome Col-0 TAIR10.1. We chose this reference because of its high quality and the richness of the associated studies [24, 38, 39].

The cumulated SVs sizes obtained for ONT and Bionano in our study are smaller than in previous studies [24, 38]. Filtering on SVs size (SVs > 1 kb) vs no size filter could explain this difference. In addition, the lack of duplications detection in ONT assembly could depend on MUMmer’s ability to detect this type of SV, reflecting the detection complexity of the duplication events, as mentioned in Goel et al (2019). In contrast, the absence of duplication detected by Bionano could be explained by polymorphic duplications between Evry.L*er*-1 maps and Col-0 TAIR10.1 reference genome, which would break the collinearity, as described in Jiao and Schneeberger (2020), and by the size of duplications (< 5 kb, [24, 64]) identified as the limit of Bionano detection.

Analyzes by the two technologies revealed a predominance of insertion, deletion and inversion with larger median and average sizes for Bionano SVs. The distribution of these types of SV is homogeneous along the chromosome arms. Most of the specific ONT SVs are located in the centromeric and pericentromeric regions. Nonetheless, a decreased coverage of the SVs in these regions was observed, in opposition previous report by Pucker et al. (2019), it can be assumed that this is probably due to technical problems such as assembly errors (for ONT SMARTdenovo) [65]. This diminution in SV coverage is also observed with Bionano technology, showing a lower density labeling in these complex regions. This contrasts previous results identifying more SVs in regions where the recombination meiotic rate decreases [24]. The filtering of SV ONTs smaller than 1 kb could again be an explanation for this contradiction. On the other hand, Bionano Solve tools well identified translocation previously characterized on Chr2 and three inversions larger than 50 kb present on Chr3 (NU_017), Chr4 (MU_097) and Chr5 (MU_138) [24, 38, 65]. For example, compared to the Col-0 TAIR10.1 reference genome, the Evry.L*er*-1 maps support a 360 kb translocation of mitochondrial sequence in the Chr2 around the 3.6 Mb Col-0 TAIR10.1 position (svID MU_153). This observation is concordant with Stupar et al. (2001) that first described the mtDNA insertion in the Col-0 TAIR10.1 reference genome [66]. In the same Chr2 region (3.29 Mbp to 3.48 Mbp, Pucker et al. (2019) identified a second 300 kb highly divergent region between *A. thaliana* Nd-1 and Col-0 TAIR10.1 reference genome. In the same study, Pucker et al. also described the lack of the entire region between 3.29 Mbp and 3.48 Mbp in L*er* reference genome, corresponding to the specific translocation of 18.2 kb detected in Evry.L*er*-1 map (svID NU_007). Zooming in this Col-0 TAIR10.1 Chr2 region (3.2 Mb to 3.5 Mb) in the Evry.L*er*-1 SDN assembly, many small contigs are observed with a missing sequence of 110 kb. This observation explains the absence of SV detection, confirming the great complexity of this region and the sequence divergence between L*er*-1 and Col-0 genome described by Pucker et al (2019). Even if the Col-0 reference genome has been improved since 2000, it remained gaps and misassemblies as largely reported in many studies [41, 56, 65, 67, 68]. A combination of the best Col-0 TAIR10.1 sequence and the new high quality optical map data obtained in this study will provide valuable resources to re-evaluate complex region assembly.

The svID MU structural variations result either from a too low density of the DLE-1 sites, or from a high divergence of genomic regions between the two ecotypes. In both cases, experimental validations are essential to validate the number and type of SVs. Nevertheless, the fact that the largest events described (MU_097 (Chr4 INV), MU_102 (Chr4 DEL), MU_153 (Chr2 ONT SVs matching the second Bionano translocation) and MU_138 (Chr5 large inversion)) were retrieved in our study, is like a proof of concept of the ONT and Bionano technologies and the parameters used for the analysis.

Comparing locations of the Evry.L*er*-1 ONT SVs with Araport11 annotations, we found that common and specific ONT SVs were preferentially linked to TE features and genes, as reported in Jiao and Schneeberger (2020). Looking at the GO-term enrichment in genes overlapping common ONT SVs, an overrepresentation in defense response and ADP-binding terms corresponding to resistance genes was observed. This result is concordant with previous studies [13, 24, 38, 69–71] in which an association between structural variations and the cluster organization of resistance genes was described.

### General conclusion

Because analyses of SV locations and their consequences heavily rely on the quality of their identification and the underlying assembly/mapping data, we aimed to compare the performance of ONT and Bionano technologies for structural variation detection. Applying stringent filters on ONT assembly mapping approach and size filters on SVs, we have shown this methodology is an easy and efficient way to detect reliable SVs. Most of the detected SVs were also identified with Bionano optical maps with high concordance despite different characteristics (average, size, median). Nevertheless, long read sequencing technologies make it possible to detect SVs more accurately, while Bionano offers a broad overview of structural rearrangements. Thereby, the choice of technology has to be based on the characteristics of the locations to be studied. If these locations are known to be gene regions without repeated sequences, the analysis of an ONT assembly will be reliable and provide more confidence in the SVs locations. Bionano’s interest will then be minimal. In contrast, if these regions are identified as being complex (areas rich in transposable elements for example) the analysis of structural variations from an ONT assembly will be more delicate since the assembly itself and the alignments of the detection will be less reliable in these locations. ONT analyzes from these regions cannot be taken at face value and will require validation (targeted experimentation by labeling, PCR, detection of these SVs by other technology, progeny analysis …). On the other hand, Bionano technology is effective in validating variation in these large complex regions. Combined with Bionano analyzes which provide an overview and point to these areas, ONT analyzes and associated results gain in value. The major limit to Bionano is the lack of access to the sequence information. In addition, whole genome SVs analyses are currently mostly limited to model organisms. However, Oxford Nanopore long reads and Bionano Genomics optical maps assemblies do not require previous knowledge on the genomic architecture or the sequence of the studied organism, this approach expands the field of suitable plant species or species complexes where in-depth SVs analyses can be performed. Unlike in animals, in plants, the heterogeneity and size of genomes, polyploidy, heterozygosity and the sequence references of species which are sometimes very different and potentially of low quality make population analyzes difficult if not impossible. Therefore, population analysis using Bionano is only possible when the reference is of very high quality and genomically very close to other ecotypes. On the other hand, these plant characteristics have less impact on the detection of variations by ONT, which is much more local with this technology.

ONT appears to be especially suitable to carry out plant population analyses and Bionano more relevant to study plasticity of genome structure, leading to an obvious complementarity of these two technologies in SVs analysis.

## Methods

### Plants

*Arabidopsis thaliana* Columbia-0 (accession number 186AV) and Landsberg *erecta*-1 (accession number 213AV) seeds were provided by the Versailles *Arabidopsis* Stock Center (National Research Institute for Agriculture, Food and Environment, Versailles, France, http://publiclines.versailles.inra.fr/). They were sown directly in soil and transplanted after 10 days. Plantlets were grown under a 16 h light/8 h night photoperiod in a growth chamber at 20 °C for 4–5 weeks. Before to harvest, the plants were dark-treated for 3 days.

### Oxford Nanopore sequencing (MinION) HMW DNA extraction

High Molecular Weight (HMW) DNA extraction was performed using a modified salting-out protocol. A total of 5 g of freshly harvested leaves was ground in liquid nitrogen with a mortar and pestle and transferred to 10 ml of 50 °C prewarmed extraction buffer in a 50 ml tube containing 1.25% SDS, 100 mM Tris-HCl, pH 8, 50 mM EDTA, 0.01% w/v PVP40. Then 37.5 μl of beta-mercaptoethanol (0.375% final) and 10 μl RNAse A (Qiagen® 100 mg/mL) were added. This solution was incubated for 30 min at 50 °C, under agitation (10 s at 300 rpm every 10 min). After incubation, 20 ml TE (10:1) were added, slowly homogenized then 10 ml of KAc 5 M. The tube was kept on ice for 5 min, then centrifuged at 4 °C during 10 min at 500 g. The solution was transferred in two 15 ml tubes and centrifuged again as previously. The supernatant was transferred in a 50 ml tube containing 1 volume of Isopropanol, slowly inverted 10 times, then centrifuged at 4 °C for 10 min at 5000 g. Pellets were washed with 20 ml ethanol 70% then centrifuged at 4 °C for 5 min at 5000 g. Supernatant was removed and pellets were not completely dried before solubilization in 100 μl of TE (10:1) prewarmed at 50 °C. The DNA solution was then incubated at 50 °C for 10 min. Field Inverted Gel Electrophoresis (Program 50–150 kb on *Pipin Pulse* from Sage Science) was used for DNA size estimation and DNA samples with molecule size above 50 kb were kept. Purity of DNA was evaluated by spectrophotometry (OD260/280 and OD260/230 ratio).

### Bionano optical maps ultra HMW DNA extraction

We performed the DNA extraction using the Base protocol n°30,068 vD (Bionano Genomics) with minor adaptations. Three grams of very young fresh leaves from each genotype were harvested from the dark-treated rosettes. The samples were placed on aluminium foil on ice then transferred to a 50 ml tube surrounded by a screened cap allowing pouring without loss of samples (Bio-Rad) The tubes were kept on ice during the nuclear isolation. Samples were treated in a fixing solution containing 2% formaldehyde under a fume hood then rinsed with fixing solution without formaldehyde. Fixed-leaves were transferred to a square Petri dish with 4 ml of Plant Homogenization Buffer plus (HB+ is HB supplemented with 1 mM spermine tetrahydrochloride, 1 mM spermidine trihydrochloride, and 0.2% 2-mercaptoethanol). Entire leaves were chopped with a razor blade in 2x2mm pieces then transferred to a new tube on ice and 7.5 ml HB+ is added. Using TissueRuptor (Qiagen) the 2x2mm pieces were blended for a total of four cycles (20 s at maximum speed then resting 30 s). Plant homogenates were filtered, first through a 100 μm then to a 40 μm cell strainer and volumes were adjusted to 45 ml. Nuclei were centrifuged at 3840 g at 4 °C during 20 min, supernatants were discarded. Nuclei were gently re-suspended in residual buffer, 3 ml of HB+ were added, then tubes were swirled on ice and the volumes were adjusted to 35 ml. Homogenates were centrifuged at 60 g at 4 °C during 3 min using minimum deceleration. Solutions were very carefully transferred to a new tube in order to avoid carry-over of debris, and filtered again through a 40 μm cell strainer. Nuclei were centrifuged at 3840 g at 4 °C during 20 min, 3 ml of HB+ were added and tubes were swirled on ice. Using Bionano Nuclei Purification by Density Gradient, nuclei homogenate was laid on the top of two solutions with different densities. After a 4500 g centrifugation at 4 °C during 40 min, the nuclei are at the interface of the two solutions. There are recovered with a wide-bore tip in about 1 ml solution and transferred in a 15 ml tube and adjusted to 14 ml with HB+. Nuclei were centrifuged at 2500 g at 4 °C during 15 min. All the buffer was removed and nuclei were re-suspended in 60 μl HB+.

The nuclei solution was adjusted to 43 °C for 3 min and melted 2% agarose from CHEF Genomic DNA Plug Kits (Bio-Rad) was added to reach a 0.82% agarose plug concentration. Plugs were cooled on aluminum blocks refrigerated on ice. Purification of the plugs was performed with Bionano Lysis Buffer adjusted to pH 9 and supplemented with proteinase K and 0.4% 2-mercaptoethanol. Plugs were digested during 2 h at 50 °C in Thermomixer then the solution was refreshed and incubated again overnight. Plugs were treated with RNAse for 1 h at 37 °C in the remaining solution. Plugs were washed three times in Wash Buffer (Bionano Genomics) then four times in TE 10:1. DNA retrieval was performed as recommended by Bionano Genomics, as follow: plugs were melted at 70 °C during 2 min then transferred immediately at 43 °C and incubated 45 min at 43 °C with 2 μl Agarase (0.5 unit/μl). The melted plugs were recovered with wide-bore tips and dialyzed on a 0.1 μm membrane disk (Millipore) floating on 10 ml TE for 1 h. DNA was quantified in triplicates with Qubit according to Bionano protocol. Two methods were used to estimate the size of DNA molecules: *Pipin Pulse* and the Qcard Argus System (Opgen) which allows DNA combing on a lane and visualization of molecules after staining under fluorescent microscope. Samples with molecules above 150 kb were kept for labeling. Protocols were performed according to Bionano Genomics with 600 ng of DNA for both Col-0 and L*er*-1 ecotypes. The direct label and stain (DLS) labeling consisted of a single enzymatic labeling reaction with DLE-1 enzyme following by DNA staining with a fluorescent marker. It was performed with 750 ng DNA. Chip loading was performed as recommended by Bionano Genomics.

### ONT sequencing (MinION) and assembly

ONT libraries were prepared according to the following protocol, using the Oxford Nanopore SQK-LSK109 kit. Genomic DNA or DNA previously fragmented to 50 kb with a Megaruptor (Diagenode S.A., Liege, Belgium) was first size-selected using a BluePippin (Sage Science, Beverly, MA, USA). The selected DNA fragments were end-repaired and 3′-adenylated with the NEBNext® Ultra™ II End Repair/dA-Tailing Module (New England Biolabs, Ipswich, MA, USA). The DNA was then purified with AMPure XP beads (Beckmann Coulter, Brea, CA, USA) and ligated with sequencing adapters provided by Oxford Nanopore Technologies (Oxford Nanopore Technologies Ltd., Oxford, UK) using Blunt/TA Ligase Master Mix (NEB). After purification with AMPure XP beads, the library was mixed with Running Buffer with Fuel Mix (ONT) and Library Loading Beads (ONT) and loaded on 4 MinION R9.4 SpotON Flow Cells per *Arabidopsis thaliana* ecotypes. The resulting FAST5 files were base-called using albacore (versions 2.1.10 and 2.3.1) and FASTA produced as described in Istace et al (2017). Canu version 1.5 (github commit ae9eecc), was used for initial read correction and trimming with the parameters minMemory = 100G, corOutCoverage = 10,000. The corrected sequences were merged in one final FASTA file per ecotype that was later used as assemblers’input.

Assemblies were performed with the relevant genome size parameter set to, or coverage calculation based on, a 130 Mb genome size. Assemblers used with default parameters were Canu version 1.5 ([54], github commit 69b5f32), Rapid Assembler (RA, [59], https://github.com/lbcb-sci/ra commit 07364a1) and SMARTdenovo version 1.0 (with the option –c 1 to run the consensus step) ([60], https://github.com/ruanjue/smartdenovo commit 61cf13d). The MUMmer suite version 3.0 [61] was run with the parameters used in Zapata et al. 2016 [38]. To analyze the assemblies, they were aligned to the reference genome of *Arabidopsis thaliana* using *nucmer* with the options -c 100 -b 500 -l 50 -g 100 -L 50. The TAIR10.1 reference genome for *A. thaliana* Columbia 0 (Col-0, GCF_000001735.4) was chosen as it is the available sequence with the latest annotation. As Pucker et al. (2019) hightlighted, the nuclear sequence is the same as the TAIR9 reference genome but chloroplastic and mitochondrial sequences were added that were necessary to detect translocation with Bionano technology. The reference genome of *Arabidopsis thaliana* Landsberg *erecta* was the one published by Zapata et al. in 2016 (L*er*, Genbank LUHQ00000000.1, [38]). The alignments were filtered with *delta-filter* (options − 1 -l 10,000 -i 0.95) and visualized with the *mummer-plot* (options --fat --large --layout –png) or DNAnexus (github commit 78e3317). These MUMmer parameters [38] allowed conserving exact matches larger than 50 bp and alignments longer than 10 kb with a minimal identity of 95%. To check assemblies completeness and fragmentation, they were compared to each other based on the metrics (Number of contigs, N50, cumulative genome sizes) and the genome alignments to the references generated with MUMmer viewed with the DNAnexus dot (https://dnanexus.github.io/dot/).

To evaluate the completeness of our ONT data, mapping of the corrected ONT reads on the Col-0 TAIR10.1 reference genome were performed with Minimap2/2.15 aligner [55] with -a -x map-ont parameters. The Samtools/1.6 depth tool with –a option [57] gave us the alignment depth at each Col-0 TAIR10.1 reference position. The error sequencing rate was inferred from the identity rate percent obtained by aligning the Evry.Col-0 and Evry.L*er*-1 trimmed corrected ONT reads on the Col-0 TAIR10.1 and L*er* reference genomes respectively.

### Bionano optical map assembly

As it can be beneficial for assembly steps, molecules sub-sampling was conducted when flowcells yielded more than 90 Gb and 600X of data. This adapted selection of molecules was made on each run with the Bionano RefAligner tool in command line (version 1.3.8041.8044 with –minlen 180 –randomize 1 –subset 1 nb_molec options) or with Bionano Access (version Solve3.3 with Filter Molecule Object utility) (Additional file 1: Tables S6 and S7).

Maps were then constructed with the tool *Generate* de novo *Assembly* of the Bionano Solve™ (Bionano Genomics, version 3.3) using the options recommended by Bionano (With pre-assembly, Non haplotype without extend and split) and a 0.115 Gb genome size. The pre-assembly step calculates noise parameters that optimize the quality of the assembly (less and larger maps). When a reference FASTA file is added, noise parameters are calculated in aligning the molecules to the reference. Otherwise, the noise parameters are estimated thanks to a first rough assembly of the molecules. For Col-0 and L*er*-1 ecotypes, three maps were obtained, one without reference, one with the Col-0 TAIR10.1 reference genome and one with the L*er* reference genome (Additional file 1: Tables S8 and S9). In our study, the metrics of these assemblies are very similar. This stability reflects that noise parameters estimated either with references fasta sequences or our data, were comparable. This is a guaranty of the quality of Bionano data and assemblies.

### ONT variation detection

Structural variations were obtained with MUMmer’s show-diff utility on the filtered alignments of SMARTdenovo assemblies against the reference genomes Col-0 TAIR10.1 and L*er*. One DIFF file per comparison was obtained. Six SV types (Gap, Duplication, Break, Jump, Inversion, Sequence) were described in the Additional file 2: Fig. S4.

### Bionano variation detection

SVs detections were performed on the optical maps built with the public reference and our SMARTdenovo ONT assemblies using the tool Convert SMAP to VCF file. VCF files were recovered, describing all the structural variations between the optical maps and the considered reference. The variations were classified into four types: deletion, insertion, translocation and inversion. SVs detection stringency is intrinsic, based on the number of aligned molecules (at least nine by default) and the number of labels across each variants breakpoint on the genome map (at least two by default) (Bionano tutorial: https://bionanogenomics.com/support-page/data-analysis-documentation/). The technology gave an interval with uncertainty about breakpoint positions (CIPOS and CIEND in VCF files). In this study, these values were used to calculate the most extended positions for the Bionano SVs and avoid the effect of label fuzz.

The low number of structural variations between Evry.Col-0 optical maps and the Col-0 TAIR10.1 reference genome (as Evry.L*er*-1 optical maps and L*er* reference genome) reflects the good collinearity between the maps and the references (Additional file 1: Table S25). SVs gave us an indication of the location of conflicts that could be due to mis-assemblies or intra-ecotype variations. Inter-ecotype detection allowed us to describe the variations between Evry.Col-0 and Evry.L*er*-1.

Quality and length characteristics were used to better describe and filter SVs. Bionano Solve associates a quality score to each INS and DEL based on sensitivity and the fraction of alternative calls in mix assemblies that were called in the alternative genome assembly [from no quality (.) or poor (0) to confident quality (20)]. We observed that this indicator follows the same trend as the SVs size (Additional file 1: Tables S11 and S16). Moreover, size range values where SVs abundances are very different between both technologies at the extremes: the smallest (< 1 kb), where ONT technology detected much more SVs and the highest (> 5 kb) where Bionano technology detected proportionally more SVs. In our comparison analysis, to remove poor quality Bionano SVs, ONT sequencing errors and high sensitivity, a filter on query SV size (> 1 kp) was applied. Confidence scores for translocation and inversion breakpoints were computed as *p*-values, giving true confidence (in Mahalanobis distance) to positive calls. The recommended cutoffs are 0.1 and 0.01 for translocation and inversion breakpoints calls respectively and were used to eliminate uncertain inversion on Chr2.

### SV description

Custom-made R and Perl scripts were used to edit other tools outputs, describe ONT and Bionano SVs (types, size), locate SVs along the chromosomes and filter them. For ONT technology, SVs identified as assemblies’discordances were quickly described and discarded before comparison. Those included sequences (SEQ), breaks (BRK) and jumps (JMP) ONT SV because they correspond to assembly or reference artifacts. Finally, size filters (more than 1 kb) were applied to take into account ONT high sequencing error rate, and low quality Bionano SVs. For Bionano SVs the largest absolute positions of the SV were conserved, taking into account the uncertainty around breakpoints due to the distance between two labels.

### SV comparison

The ONT and Bionano SV medians sizes comparison was dealed with the ggpubr R package (http://www.sthda.com/english/wiki/unpaired-two-samples-wilcoxon-test-in-r). The boxplots were drawn with *ggboxplot* tool (my_data, x = “Technology”, y = “size”, fill = “Technology”, palette = c(“darkgoldenrod1”, “darkorchid”), notch = TRUE, ylab = “Size in base (log10)”, xlab = FALSE, ylim = c(0,7), add = “median”, add.params = list (size = 0.5,color = “red”)) and the Wilcoxon statistical test (H0 = median of ONT SV size is less than Bionano one) performed with *stat_compare_means* tool (method = “wilcox.test”, methods.args = list (alternative = “less”), label.y = 6). The test is considered as statistical when the p-value is less than the selected threshold. We chose here the standard one of 5%.

Comparison of SV obtained with both ONT and Bionano technologies were based on the overlap of their absolute positions.

ONT SV and Bionano SVs files were used after conversion to BED format to identify overlapping regions with BEDtools (version 2.27.1, github commit cd82ed5, “bedtools intersect -wa -wb -a INPUT1.bed -b INPUT2.bed -loj > OUTPUT.bed”). Raw comparisons were then compared, compiled and formatted in one final output file using custom-made R scripts. For each SVs location, this file contained descriptors (SVs size, type, quality) for both technologies, information on the type of conflict and a 2 letter code. This code characterized the SVs location as follows: the first letter corresponds to the ONT SV characterization, the second to the Bionano SV. M (“Multiple”) means more than one SV locations, U (“Unique”) one SV location, N (“No”) no SV location. For example, the code “MU” means that this location harbored multiple ONT SV corresponding to a unique Bionano location. No UM localization (corresponding to an ONT localization overlapping several Bionano SV localizations) was detected in our study. The landscapes and SVs occurrences visualization was performed with Circos/0.69.9 tool (perl/5.16.3 [72]).

### SV and annotation

SVs overlapping a gene and/or TE were identified with the bedtools intersect by comparing their absolute positions to *A. thaliana* Col-0 annotations (July 11th 2019 release, TAIR10_GFF3_genes_transposons.gff). Lists of genes impacted by SV for both technologies were extracted and a GO-term enrichment analysis performed using Fisher’s Exact test with a Bonferroni correction in PANTHER (released 20,200,407 with GO Ontology database DOI: 10.5281/zenodo.3873405 Released 2020-06-01, [63], http://go.pantherdb.org/). Significance was evaluated based on a *P*-value ≤10–5 and an FDR value ≤0.01 [73].

## Supplementary Information

**Additional file 1.**

**Additional file 2.**

## Data Availability

The ONT reads files and the Bionano molecules files have been submitted to the European Nucleotide Archive (http://www.ebi.ac.uk) and are publicly available with the accession numbers PRJEB44307 and ERZ1959921 respectively. Assemblies and optical maps of the Evry.Col-0 and Evry.L*er*-1 genomes are publicly available in separated ENA studies with the accession number ERS6258851 and ERS6258852 respectively. “Custom-made” scripts of this study are deposited in the INRAE repository https://forgemia.inra.fr/epgv/sv-arabido and publicly available..

## Abbreviations

bp: Base pairs
BRK: Break
CGH: Comparative Genomic Hybridization
CNV: Copy number variations
Col-0: *Arabidopsis thaliana* ecotypes Columbia-0
DEL: Deletion
DLE-1: Direct Label Enzyme – 1
DLS: Direct Label and Stain
DUP: Duplication
Hi-C: HIgh-throughput chromatin conformation Capture
Indels: Insertions/deletions
INS: Insertion
INV: Inversion
JMP: Jump
Kb: Kilobases
L*er*-1: *Arabidopsis thaliana* ecotypes Landsberg *erecta* 1
NA: Not Available
NGS: Next Generation Sequence
ONT: Oxford Nanopore Technologies
PAV: Presence/absence variations
RA: Rapid Assembler
SDN: SMARTdenovo
SEQ: Sequence
SNP: Single Nucleotid Polymorphism
SV: Structural Variation
TAIR: TAIR10.1 last version of *Arabdopsis thaliana* Col-0 reference sequence genome available at the The Arabidopsis Information Resource repository
TE: Transposable Element
TRA: Translocation

## References

[1] Saxena RK, Edwards D, Varshney RK (2014). Structural variations in plant genomes. Brief Funct Genom.

[2] Escaramís G, Docampo E, Rabionet R (2015). A decade of structural variants: description, history and methods to detect structural variation. Brief Funct Genomics..

[3] Zhang X, Chen X, Liang P, Tang H. Cataloging plant genome structural variations. Curr Issues Mol Biol. 2018:27:181–94. 10.21775/cimb.027.181 28885182

[4] Ho SS, Urban AE, Mills RE (2020). Structural variation in the sequencing era. Nat Rev Genet.

[5] Wendel JF, Jackson SA, Meyers BC, Wing RA (2016). Evolution of plant genome architecture. Genome Biol.

[6] Gabur I, Chawla HS, Snowdon RJ, Parkin IAP (2019). Connecting genome structural variation with complex traits in crop plants. Theor Appl Genet.

[7] Schiessl S-V, Katche E, Ihien E, Chawla HS, Mason AS (2019). The role of genomic structural variation in the genetic improvement of polyploid crops. Crop Journal.

[8] Voichek Y, Weigel D (2020). Identifying genetic variants underlying phenotypic variation in plants without complete genomes. Nat Genet.

[9] Muñoz-Amatriaín M, Eichten SR, Wicker T, Richmond TA, Mascher M, Steuernagel B (2013). Distribution, functional impact, and origin mechanisms of copy number variation in the barley genome. Genome Biol.

[10] Dolatabadian A, Patel DA, Edwards D, Batley J (2017). Copy number variation and disease resistance in plants. Theor Appl Genet.

[11] Fuentes RR, Chebotarov D, Duitama J, Smith S, De la Hoz JF, Mohiyuddin M (2019). Structural variants in 3000 rice genomes. Genome Res.

[12] Tao Y, Zhao X, Mace E, Henry R, Jordan D (2019). Exploring and exploiting Pan-genomics for crop improvement. Mol Plant.

[13] Wei H, Liu J, Guo Q, Pan L, Chai S, Cheng Y (2020). Genomic organization and comparative phylogenic analysis of NBS-LRR resistance gene family in Solanum pimpinellifolium and Arabidopsis thaliana. Evol Bioinformatics Online.

[14] Prunier J, Caron S, MacKay J (2017). CNVs into the wild: screening the genomes of conifer trees (Picea spp.) reveals fewer gene copy number variations in hybrids and links to adaptation. BMC Genomics.

[15] Prunier J, Giguère I, Ryan N, Guy R, Soolanayakanahally R, Isabel N (2019). Gene copy number variations involved in balsam poplar ( Populus balsamifera L.) adaptive variations. Mol Ecol.

[16] Wang Y, Xiong G, Hu J, Jiang L, Yu H, Xu J (2015). Copy number variation at the GL7 locus contributes to grain size diversity in rice. Nat Genet.

[17] Gong C, Du Q, Xie J, Quan M, Chen B, Zhang D. Dissection of Insertion–Deletion Variants within Differentially Expressed Genes Involved in Wood Formation in Populus. Front Plant Sci [Internet]. 2018; [cited 2019 Aug 20];8. Available from: https://www.frontiersin.org/articles/10.3389/fpls.2017.02199/full?report=reader. 10.3389/fpls.2017.02199 PMC5778123 29403506

[18] Gao L, Gonda I, Sun H, Ma Q, Bao K, Tieman DM (2019). The tomato pan-genome uncovers new genes and a rare allele regulating fruit flavor. Nat Genet.

[19] Tranchant-Dubreuil C, Rouard M, Sabot F. Plant pangenome: impacts on phenotypes and evolution. Ann Plant Rev. 2019; May [cited 2021 Feb 11]; Available from: https://hal.archives-ouvertes.fr/hal-02053647.

[20] Khan AW, Garg V, Roorkiwal M, Golicz AA, Edwards D, Varshney RK (2020). Super-Pangenome by integrating the wild side of a species for accelerated crop improvement. Trends Plant Sci.

[21] Li R, Li Y, Zheng H, Luo R, Zhu H, Li Q (2010). Building the sequence map of the human pan-genome. Nat Biotechnol.

[22] Sherman RM, Forman J, Antonescu V, Puiu D, Daya M, Rafaels N (2019). Assembly of a pan-genome from deep sequencing of 910 humans of African descent. Nat Genet.

[23] Duan Z, Qiao Y, Lu J, Lu H, Zhang W, Yan F (2019). HUPAN: a pan-genome analysis pipeline for human genomes. Genome Biol.

[24] Jiao W-B, Schneeberger K (2020). Chromosome-level assemblies of multiple Arabidopsis genomes reveal hotspots of rearrangements with altered evolutionary dynamics. Nat Commun.

[25] Song J-M, Guan Z, Hu J, Guo C, Yang Z, Wang S (2020). Eight high-quality genomes reveal pan-genome architecture and ecotype differentiation of Brassica napus. Nat Plants.

[26] Springer NM, Ying K, Fu Y, Ji T, Yeh C-T, Jia Y (2009). Maize Inbreds exhibit high levels of copy number variation (CNV) and presence/absence variation (PAV) in genome content. PLoS Genet.

[27] Swanson-Wagner RA, Eichten SR, Kumari S, Tiffin P, Stein JC, Ware D (2010). Pervasive gene content variation and copy number variation in maize and its undomesticated progenitor. Genome Res.

[28] Hwang JE, Kim S-H, Jung IJ, Han SM, Ahn J-W, Kwon S-J, et al. Comparative genomic hybridization analysis of rice dwarf mutants induced by gamma irradiation. Genet Mol Res. 2016;15(4):gmr15049092. 10.4238/gmr15049092 28081277

[29] Mabire C, Duarte J, Darracq A, Pirani A, Rimbert H, Madur D (2019). High throughput genotyping of structural variations in a complex plant genome using an original Affymetrix® axiom® array. BMC Genomics.

[30] Redmond SN, Sharma A, Sharakhov I, Tu Z, Sharakhova M, Neafsey DE (2020). Linked-read sequencing identifies abundant microinversions and introgression in the arboviral vector Aedes aegypti. BMC Biol.

[31] Rhoads A, Au KF (2015). PacBio sequencing and its applications. Genom Proteomics Bioinform.

[32] Lu H, Giordano F, Ning Z (2016). Oxford Nanopore MinION sequencing and genome assembly. Genom Proteomics Bioinform.

[33] Mahmoud M, Gobet N, Cruz-Dávalos DI, Mounier N, Dessimoz C, Sedlazeck FJ (2019). Structural variant calling: the long and the short of it. Genome Biol.

[34] Huddleston J, Chaisson MJP, Steinberg KM, Warren W, Hoekzema K, Gordon D (2017). Discovery and genotyping of structural variation from long-read haploid genome sequence data. Genome Res.

[35] De Coster W, D’Hert S, Schultz DT, Cruts M, Van Broeckhoven C, Berger B (2018). NanoPack: visualizing and processing long-read sequencing data. Bioinformatics..

[36] Chaisson MJP, Sanders AD, Zhao X, Malhotra A, Porubsky D, Rausch T (2019). Multi-platform discovery of haplotype-resolved structural variation in human genomes. Nat Commun.

[37] Beyter D, Ingimundardottir H, Oddsson A, Eggertsson HP, Bjornsson E, Jonsson H (2021). Long-read sequencing of 3,622 Icelanders provides insight into the role of structural variants in human diseases and other traits. Nat Genet.

[38] Zapata L, Ding J, Willing E-M, Hartwig B, Bezdan D, Jiao W-B (2016). Chromosome-level assembly of *Arabidopsis thaliana* L *er* reveals the extent of translocation and inversion polymorphisms. Proc Natl Acad Sci.

[39] Michael TP, Jupe F, Bemm F, Motley ST, Sandoval JP, Lanz C (2018). High contiguity Arabidopsis thaliana genome assembly with a single nanopore flow cell. Nat Commun.

[40] Jupe F, Rivkin AC, Michael TP, Zander M, Motley ST, Sandoval JP, et al. The complex architecture and epigenomic impact of plant T-DNA insertions. PLoS Genet [Internet]. 2019;15(1) Jan 18 [cited 2021 May 20]. Available from: https://www.ncbi.nlm.nih.gov/pmc/articles/PMC6338467/. 10.1371/journal.pgen.1007819 PMC6338467 30657772

[41] Pucker B, Kleinbölting N, Weisshaar B. Large scale genomic rearrangements in selected *Arabidopsis thaliana* T-DNA lines are caused by T-DNA insertion mutagenesis [internet]. Plant Biol. 2021; Mar [cited 2021 Mar 8]. Available from: http://biorxiv.org/lookup/doi/10.1101/2021.03.03.433755. 10.1186/s12864-021-07877-8 PMC8348815 34362298

[42] Belser C, Istace B, Denis E, Dubarry M, Baurens F-C, Falentin C (2018). Chromosome-scale assemblies of plant genomes using nanopore long reads and optical maps. Nature Plants.

[43] Sun S, Zhou Y, Chen J, Shi J, Zhao H, Zhao H (2018). Extensive intraspecific gene order and gene structural variations between Mo17 and other maize genomes. Nat Genet.

[44] Dumschott K, Schmidt MH-W, Chawla HS, Snowdon R, Usadel B (2020). Oxford Nanopore sequencing: new opportunities for plant genomics? Raines C, editor. J Exp Bot.

[45] Lam ET, Hastie A, Lin C, Ehrlich D, Das SK, Austin MD (2012). Genome mapping on nanochannel arrays for structural variation analysis and sequence assembly. Nat Biotechnol.

[46] Cao H, Hastie AR, Cao D, Lam ET, Sun Y, Huang H (2014). Rapid detection of structural variation in a human genome using nanochannel-based genome mapping technology. GigaSci..

[47] Levy-Sakin M, Pastor S, Mostovoy Y, Li L, Leung AKY, McCaffrey J (2019). Genome maps across 26 human populations reveal population-specific patterns of structural variation. Nat Commun.

[48] Leung AK-Y, Liu MC-J, Li L, Lai YY-Y, Chu C, Kwok P-Y, et al. OMMA enables population-scale analysis of complex genomic features and phylogenomic relationships from nanochannel-based optical maps. Gigascience. 2019;8(7) 1 [cited 2019 Sep 24]. Available from: https://academic.oup.com/gigascience/article/8/7/giz079/5530323. 10.1093/gigascience/giz079 PMC6615982 31289833

[49] Soto DC, Shew C, Mastoras M, Schmidt JM, Sahasrabudhe R, Kaya G, et al. Identification of structural variation in chimpanzees using optical mapping and Nanopore sequencing. Genes (Basel). 2020;11(3):276. 10.3390/genes11030276 PMC7140787 32143403

[50] Yuan Y, Milec Z, Bayer PE, Vrána J, Doležel J, Edwards D (2018). Large-scale structural variation detection in subterranean clover subtypes using optical mapping. Front Plant Sci.

[51] Maestri S, Gambino G, Minio A, Perrone I, Cosentino E, Giovannone B, et al. Genomic structural variation in ‘Nebbiolo’ grapevines at the individual, clonal and cultivar levels. bioRxiv. 2020. 10.1101/2020.10.27.357046.

[52] Dixon JR, Xu J, Dileep V, Zhan Y, Song F, Le VT (2018). Integrative detection and analysis of structural variation in cancer genomes. Nat Genet.

[53] Long E, Evans C, Chaston J, Udall JA (2018). Genomic structural variations within five continental populations of *Drosophila melanogaster*. G3 (Bethesda).

[54] Koren S, Walenz BP, Berlin K, Miller JR, Bergman NH, Phillippy AM (2017). Canu: scalable and accurate long-read assembly via adaptive *k* -mer weighting and repeat separation. Genome Res.

[55] Li H (2018). Minimap2: pairwise alignment for nucleotide sequences. Bioinformatics..

[56] Sloan DB, Wu Z, Sharbrough J (2018). Correction of persistent errors in Arabidopsis reference mitochondrial genomes. Plant Cell.

[57] Danecek P, Bonfield JK, Liddle J, Marshall J, Ohan V, Pollard MO, Whitwham A, Keane T, SA MC, Davies RM, Li H (2021). Twelve years of SAMtools and BCFtools. GigaScience.

[58] Toda N, Rustenholz C, Baud A, Le Paslier M-C, Amselem J, Merdinoglu D, et al. NLGenomeSweeper: A Tool for Genome-Wide NBS-LRR Resistance Gene Identification. Genes (Basel). 2020;11(3) 20 [cited 2021 Apr 12]. Available from: https://www.ncbi.nlm.nih.gov/pmc/articles/PMC7141099/. 10.3390/genes11030333 PMC7141099 32245073

[59] Vaser R (2018). Rapid Assembler.

[60] Liu H, Wu S, Li A, Ruan J. SMARTdenovo: a de novo assembler using long noisy reads. Gigabyte. 2021. 10.46471/gigabyte.15. 10.46471/gigabyte.15 PMC9632051 36824332

[61] Kurtz S, Phillippy A, Delcher AL, Smoot M, Shumway M, Antonescu C, et al. Versatile and open software for comparing large genomes. Genome Biol. 2004;5(2):R12. 10.1186/gb-2004-5-2-r12. 10.1186/gb-2004-5-2-r12 PMC395750 14759262

[62] Jain M, Tyson J, Loose M, Ip C, Eccles D, O’Grady J (2017). MinION analysis and reference consortium: phase 2 data release and analysis of R9.0 chemistry. F1000Research..

[63] Mi H, Muruganujan A, Ebert D, Huang X, Thomas PD (2019). PANTHER version 14: more genomes, a new PANTHER GO-slim and improvements in enrichment analysis tools. Nucleic Acids Res.

[64] Goel M, Sun H, Jiao W-B, Schneeberger K (2019). SyRI: finding genomic rearrangements and local sequence differences from whole-genome assemblies. Genome Biol.

[65] Pucker B, Holtgräwe D, Stadermann KB, Frey K, Huettel B, Reinhardt R (2019). A chromosome-level sequence assembly reveals the structure of the Arabidopsis thaliana Nd-1 genome and its gene set. PLoS One.

[66] Stupar RM, Lilly JW, Town CD (2001). Complex mtDNA constitutes an approximate 620-kb insertion on Arabidopsis thaliana chromosome 2: implication of potential sequencing errors caused by large-unit repeats. PNAS..

[67] Initiative TAG (2000). Analysis of the genome sequence of the flowering plant Arabidopsis thaliana. Nature..

[68] Zmienko A, Marszalek-Zenczak M, Wojciechowski P, Samelak-Czajka A, Luczak M, Kozlowski P (2020). AthCNV: a map of DNA copy number variations in the Arabidopsis genome [OPEN]. Plant Cell.

[69] Meyers BC, Kozik A, Griego A, Kuang H, Michelmore RW (2003). Genome-wide analysis of NBS-LRR-encoding genes in Arabidopsis. Plant Cell.

[70] Song Y, Ling N, Ma J, Wang J, Zhu C, Raza W (2016). Grafting resulted in a distinct proteomic profile of watermelon root exudates relative to the un-grafted watermelon and the rootstock plant. J Plant Growth Regul.

[71] Staal J, Kaliff M, Bohman S, Dixelius C (2006). Transgressive segregation reveals two Arabidopsis TIR-NB-LRR resistance genes effective against Leptosphaeria maculans, causal agent of blackleg disease. Plant J.

[72] Krzywinski M, Schein J, Birol I, Connors J, Gascoyne R, Horsman D (2009). Circos: an information aesthetic for comparative genomics. Genome Res.

[73] Lin M, Fang J, Hu C, Qi X, Sun S, Chen J (2020). Genome-wide DNA polymorphisms in four Actinidia arguta genotypes based on whole-genome re-sequencing. PLoS One.

