# Supplementary material for: Oxford Nanopore and Bionano Genomics technologies evaluation for plant structural variation detection
Source: BMC Genomics. 2022 Apr 21;23:317. doi: 10.1186/s12864-022-08499-4 (PMC9026655; doi:10.1186/s12864-022-08499-4)

Figure S1. Views of Evry.Col-0 contigs alignments against Col-0 TAIR10.1 reference genome (dotted end).

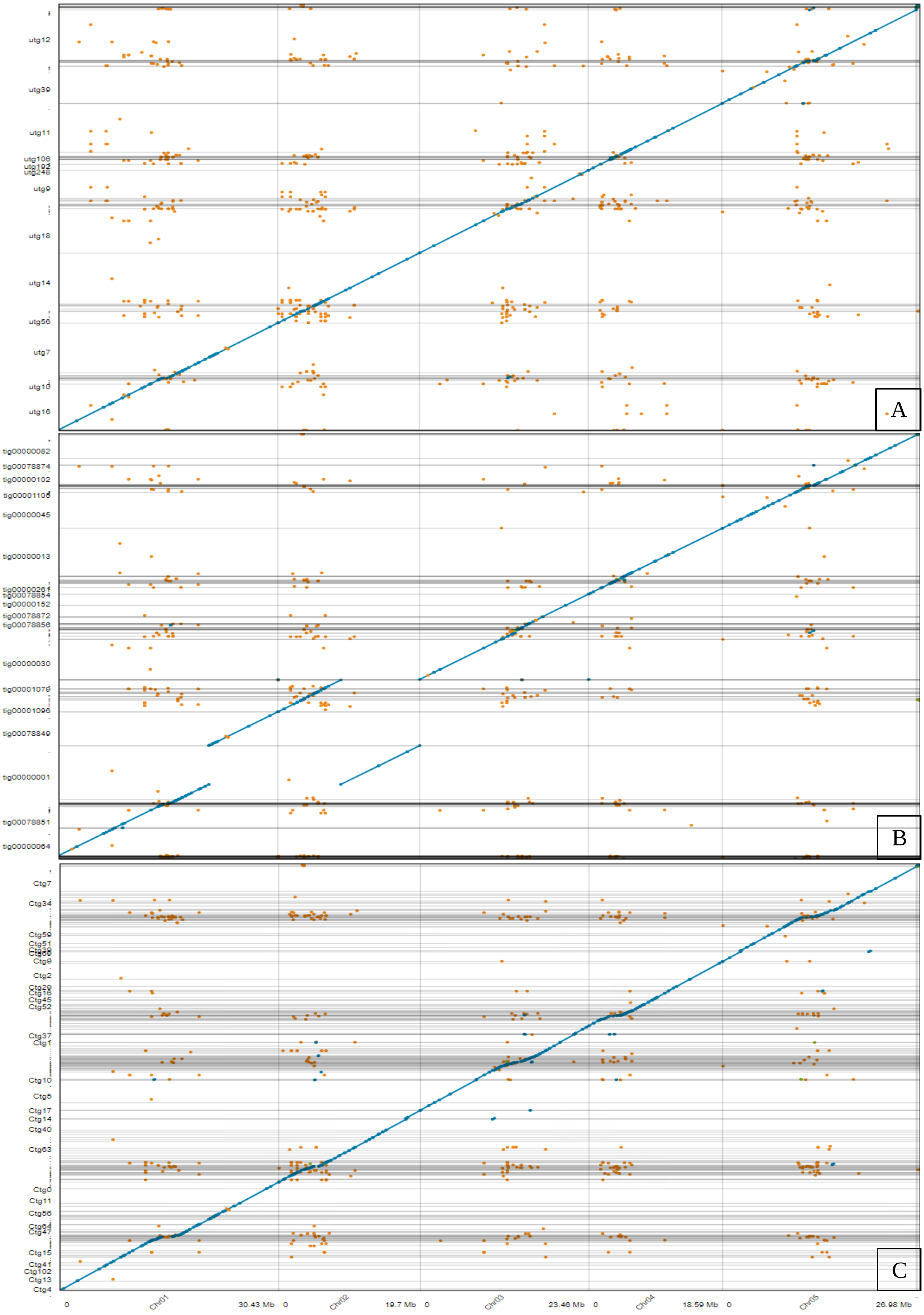

Figure S2. Views of Evry.Ler-1 contigs alignments on Ler reference genome (dotted end).

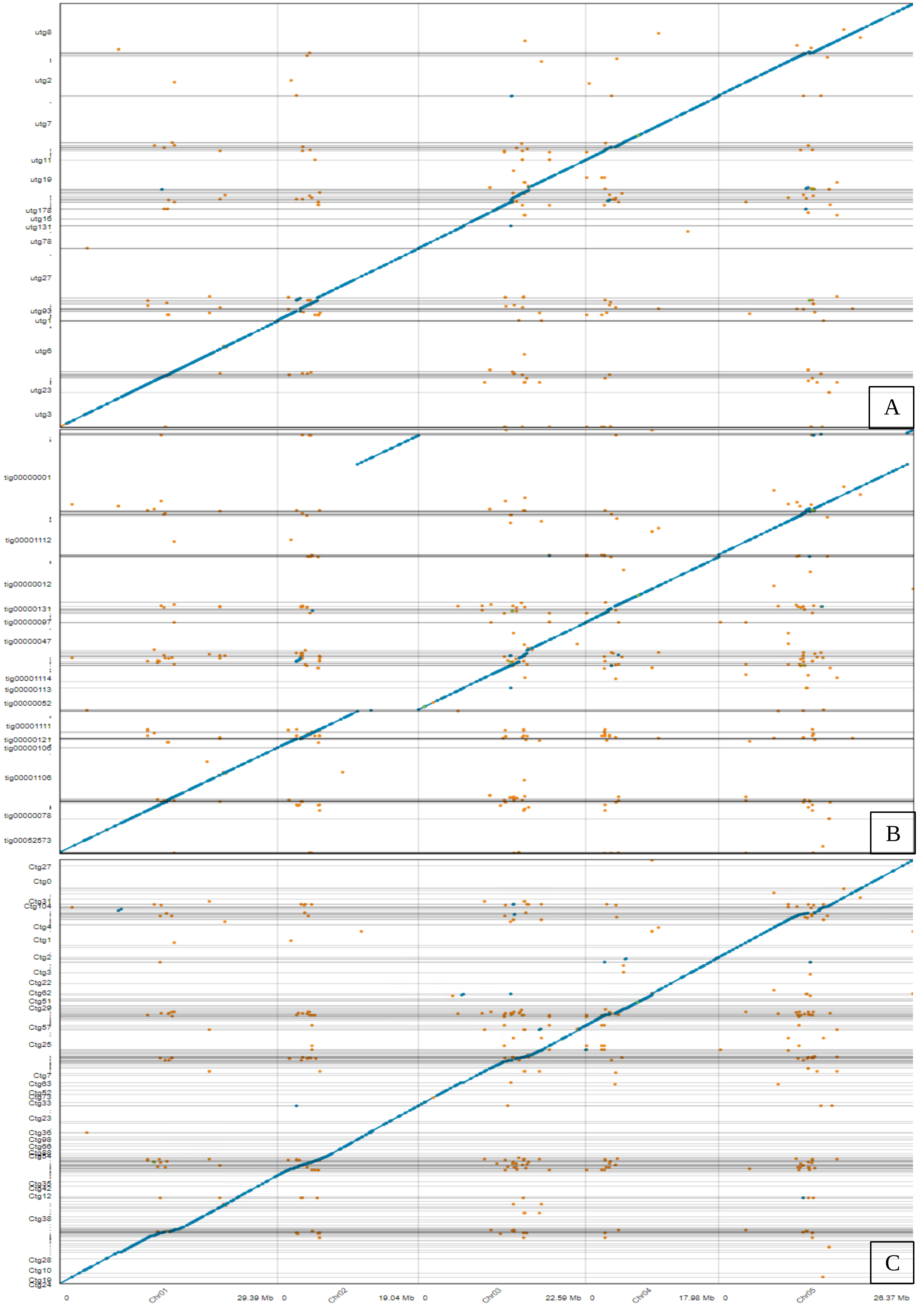

Figure S3. Bionano Access view of Evry.L*er*-1 maps aligned on Col-0 TAIR10.1 reference genome.

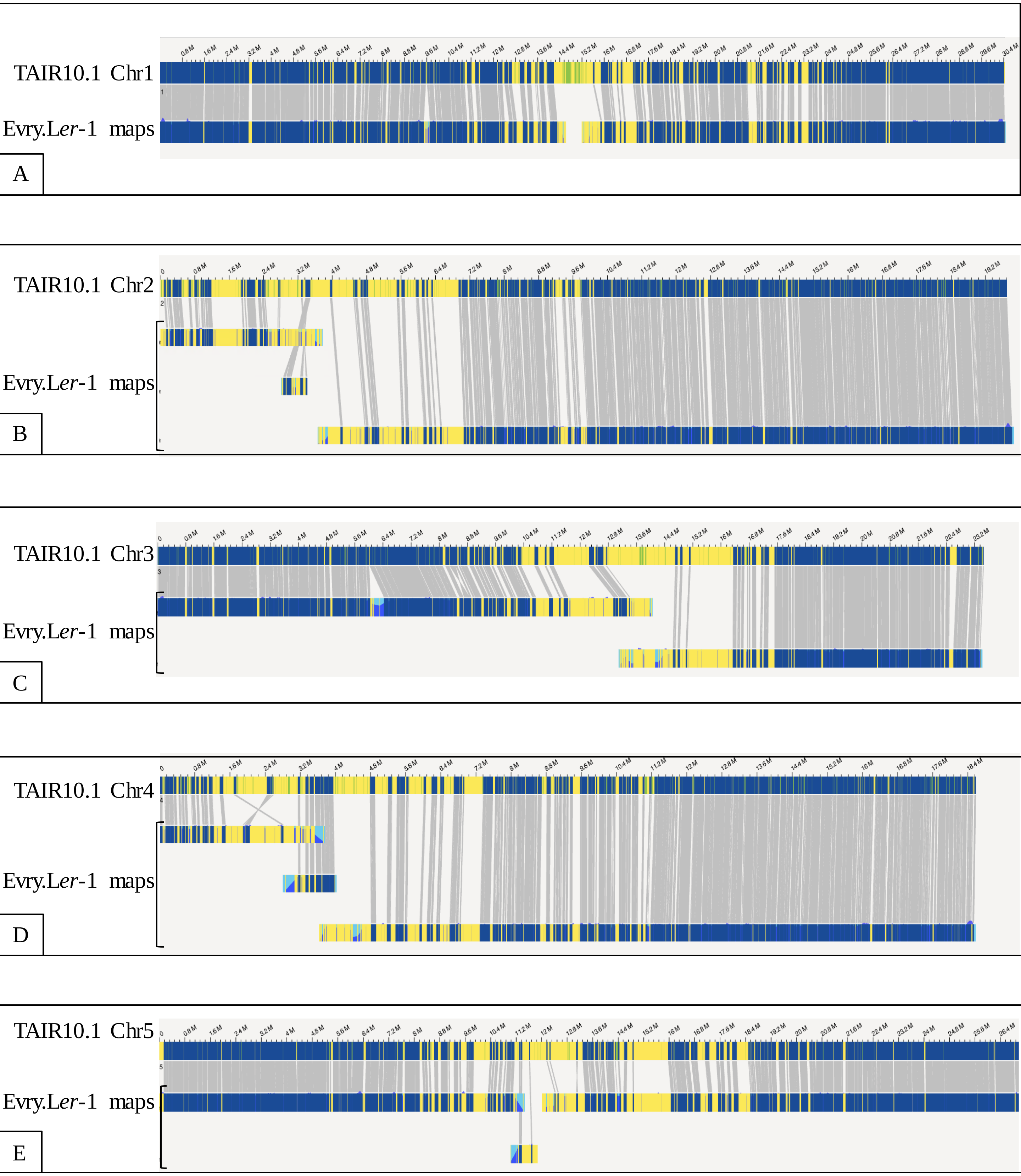

Figure S4. Description of SVs detected by MUMmer show-diff and Bionano Access tools.

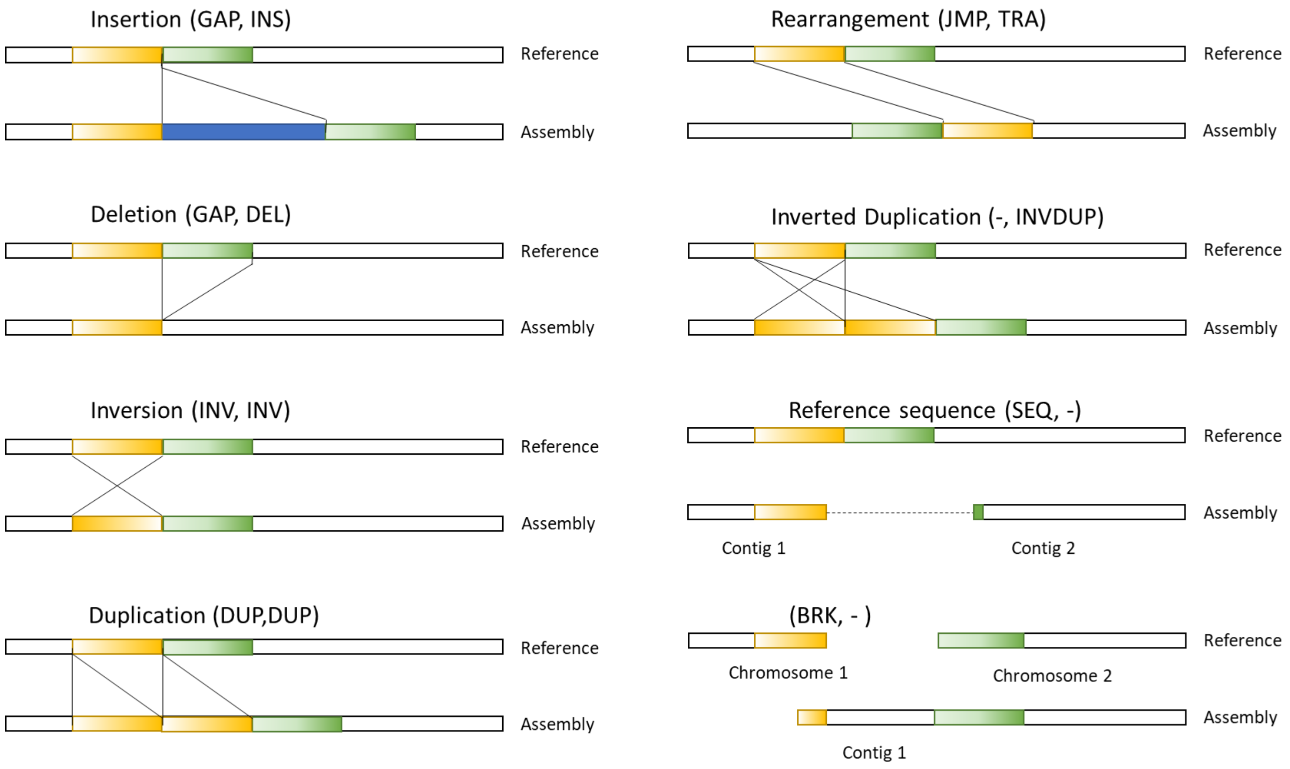

Figure S5. Evry.Col-0 SVs (>1kb) occurrences and landscape of L*er* reference chromosomes.

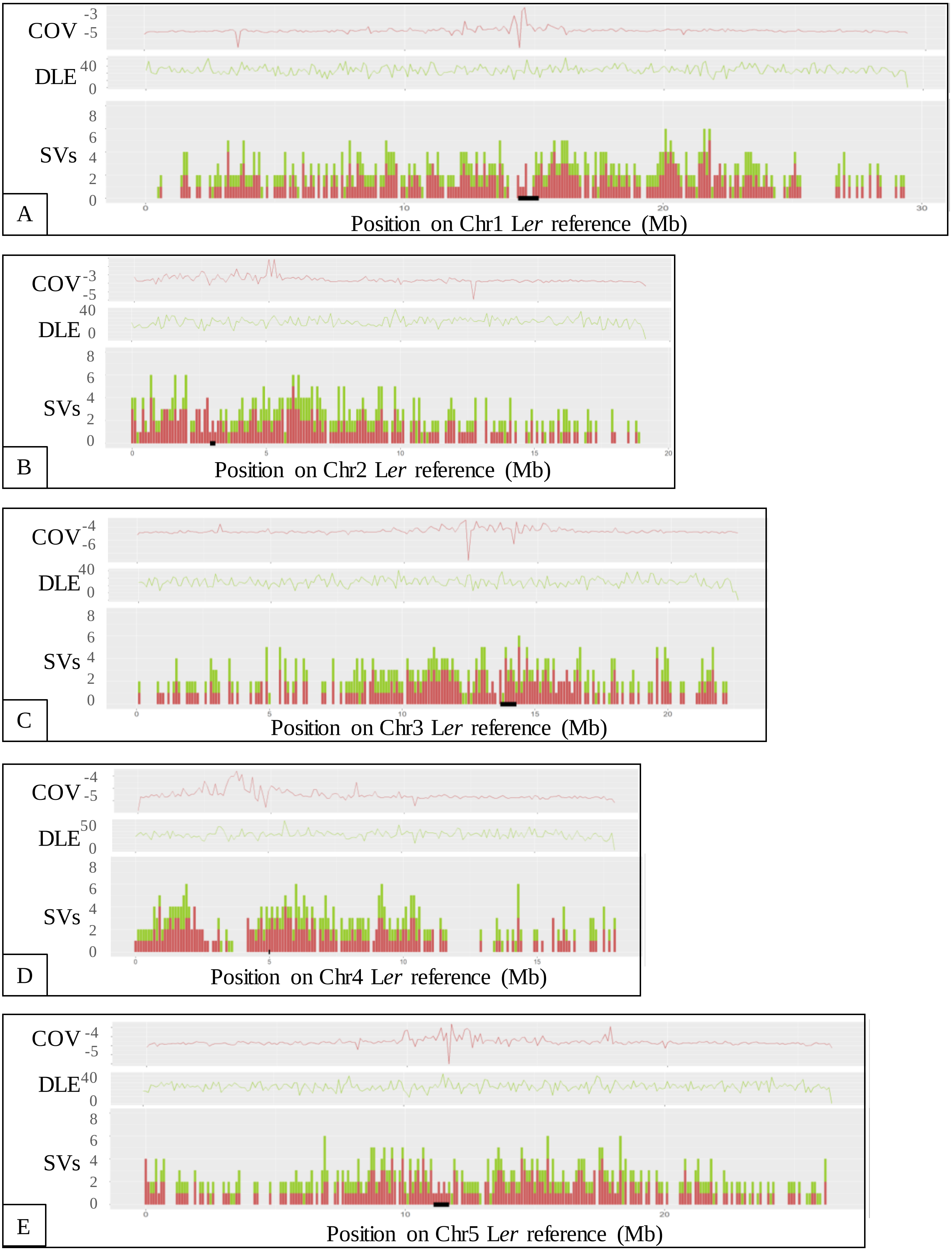

Supplement: Supplementary file 2 — Additional file 2. [file 12864_2022_8499_MOESM2_ESM.pdf]
